# Correlation of Myopia with Physical Exercise and Sleep Habits among Suburban Adolescents

**DOI:** 10.1155/2020/2670153

**Published:** 2020-07-25

**Authors:** Yuan Qu, Jianfeng Yu, Wei Xia, Huijuan Cai

**Affiliations:** ^1^Department of Ophthalmology, Zhujiajiao People's Hospital of Qingpu District, Shanghai, China; ^2^Department of Ophthalmology, Zhongshan Hospital Qingpu Branch, Shanghai, China; ^3^Department of Physical Examination, Zhujiajiao People's Hospital of Qingpu District, Shanghai, China

## Abstract

**Purpose:**

To analyse the correlation of myopia with physical exercise and sleep habits to explore the possible risk or protective factors of juvenile myopia.

**Methods:**

This study was a retrospective cross-sectional study. A total of 1831 students aged 11–18 years from five schools in Qingpu completed questionnaires from 2018 to 2019; the students were divided into the nonmyopia group and the myopia group, with students in the myopia group having myopia in at least one eye. Based on the chi-square test, the variables with statistical significance were selected for the logistic regression analysis. Adjusted odds ratios (ORs) and 95% confidence intervals (CIs) were determined.

**Results:**

Myopia was positively associated with older age (OR = 2.216, 95% CI: 1.720–2.855), having a parent with myopia (father: OR = 2.463, 95% CI: 1.900–3.193; mother: OR = 2.853, 95% CI: 2.232–3.646), and a late bedtime during holidays (before 10 pm: *p* = 0.002; 10 pm-11 pm: OR = 1.516, 95% CI: 1.095–2.100; 11 pm-12 am: OR = 1.966, 95% CI: 1.383–2.795), but negatively correlated with male gender (OR = 0.693, 95% CI: 0.549–0.876), a late daily wake time (OR = 0.782, 95% CI: 0.621–0.985), and having more days per week of outdoor activities during the semester (OR = 0.764, 95% CI: 0.597–0.978). After adjustment for demographic characteristics, myopia was positively associated with a late bedtime during holidays (before 10 pm: *p* = 0.003; 10 pm-11 pm: OR = 1.570, 95% CI: 1.076–2.289; 11 pm-12 am: OR = 2.173, 95% CI: 1.451–3.253; after 12 am: OR = 1.786, 95% CI: 1.093–2.919), but negatively correlated with a late daily wake time during the semester (OR = 0.761, 95% CI: 0.581–0.997).

**Conclusion:**

The association of outdoor exercise with myopia among Chinese suburban adolescents does not seem to be as strong as that of sleep habits. Future research could seek to provide consistent evidence for a potential association between myopia and sleep habits.

## 1. Introduction

Myopia is a major vision disorder that typically onsets during adolescents. It has been listed by the World Health Organization (WHO) as one of the five ocular diseases that must be improved or eliminated in “Vision 2020: the Right to Sight” [1]. Because of its widespread occurrence in student populations and effects on not only physical health but also the risk of complications of the eye, including myopic retinopathy, retinal detachments, and blindness, myopia has become the focus of many studies [2–5].

To explore which factors are related to adolescent myopia, researchers around the world have studied a series of possible related factors to intervene in myopia in adolescence. A number of studies have shown that parental myopia, long-term near work, and lack of outdoor activities are risk factors for myopia [6–10]. Some studies [8–10] have noted that controlling near working time and prolonging the outdoor activity time may prevent the progression of myopia in adolescents. Nevertheless, due to some factors that cannot be controlled by teenagers, such as air pollution and severe weather, e.g., long-term snowstorms or rainy seasons in some areas, long-term outdoor activities may be unrealistic. Other controllable related factors, such as the type of sports or engagement in indoor or outdoor physical activities, can also be discussed. The types of sports are common sports that students can participate in everyday and not professional-level sports, such as running, cycling, and other activities carried out in school sports classes. In addition, sleep duration was found to be inversely related to myopia in adolescents. A short sleep duration was also associated with juvenile myopia [11–13]. Studies could also examine whether teenagers' different sleep habits, such as bedtime, wake time, and mid-day naps, are a risk factor or protective factor for myopia.

Therefore, the purpose of this study was to explore the correlation of myopia with physical exercise and sleep among adolescents to provide suggestions for interventions for juvenile myopia.

## 2. Materials and Methods

### 2.1. Study Design

This study was a retrospective cross-sectional study. It followed the tenets of the Declaration of Helsinki and was approved by the Ethics Committee of Zhujiajiao People's Hospital, Qingpu Health Commission, and Qingpu Science and Technology Development Foundation in Shanghai (No. QKY2018-22). Students from two junior high schools (grades 7–8, aged 11–14), two senior high schools (grades 1–2, aged 15–18), and one vocational school (aged 15–18) in Qingpu participated. After the nature of the study was explained to students, teachers, and parents, written informed consent was obtained from at least one parent, and verbal agreement to participate in the study was obtained from the student. We investigated the correlation of myopia with physical exercise and sleep using a questionnaire.

Qingpu is a suburb of Shanghai. Every year, the physical examination department of our hospital performs student medical examinations for some schools in Qingpu District, in which schools are managed by the Qingpu Education Commission and Qingpu Health Commission. Our hospital has been responsible for student medical examinations for two junior high schools, two senior high schools, and one vocational school for the past five years; the research participants in this study were studying in these schools. The ophthalmologist and assistants in the physical examination department were responsible for the eye examination. The school doctors in these schools assisted in the distribution and collection of questionnaires. One ophthalmologist screened the questionnaires and entered them into a computer, and the other ophthalmologist checked the data from the computer against the answers to the questionnaire.

### 2.2. Participants

All available students in the selected classes in 2018 and 2019 were invited to participate in our study. The exclusion criteria were as follows: (1) simple hyperopia or astigmatism with uncorrected visual acuity less than 1.0; (2) any ophthalmological or systemic disease that could have affected visual acuity (amblyopia, glaucoma, uveitis, retinopathy, or optic neuropathy); (3) high myopia, which was defined as ≤−6.0 diopters (D); and (4) myopia with best-corrected visual acuity (BCVA) or glasses-corrected visual acuity less than 1.0.

Through the annual student medical examination starting in kindergarten, if abnormal vision is found, parents are notified that they should accompany their children to the hospital for a series of eye examinations such as an optometry examination to determine the cause. Therefore, most of the 11- to 18-year-old students who participated in the study would know their refractive status. In addition, the questionnaire was distributed after the most recent student medical examination that included visual examination, including for uncorrected visual acuity and glasses-corrected visual acuity. The questionnaire presented options for the refractive range from which participants could choose. According to the questionnaire responses, the participants were divided into two groups: the nonmyopia group and the myopia group consisting of the students with myopia in at least one eye.

### 2.3. Questionnaire

The doctors from our physical examination department gave the questionnaires and written informed consent forms to the school doctors on the first day of the student medical examination, and the school doctors assisted in their distribution. The parents obtained and signed written informed consent forms. Meanwhile, the students completed the detailed questionnaire within one week. Then, the school doctors collected the questionnaires and gave them to us.

Information on sociodemographic factors was collected, including gender, age, grade level, school, and parental myopia. Parental myopia was assessed by asking the following question for each student: “Is your father (or mother) myopic?” The response categories were “yes” and “no”.

Some studies have noted that juvenile myopia is related to the outdoor activity time, and that short sleep duration is also associated with myopia in adolescents. In our study, in addition to outdoor activities and sleep duration, we also compared time spent on indoor activities and sleep habits. Adolescents' physical exercise was assessed by asking the following questions: (1) How long do you spend on outdoor activities during every activity session and every week? (2) How many days do you participate in outdoor activities every week? (3) How much time do you spend on indoor activities every week? and (4) What kinds of sports do you usually do (select up to three items)? The assessment of sleep habits mainly focused on the following questions: (1) Do you have a habit of taking mid-day naps? (2) How long do you sleep every night on average? and (3) What time do you get up and go to bed everyday on average? All of the above questions distinguished between the semester (including weekdays during the semesters and school days, i.e., the days when the students needed to attend class) and holidays (including weekends and various vacations, i.e., the days when the students could arrange their time freely without attending school) (S1).

The visual acuity assessment was based on the last student medical examination. The questionnaire presented options for the refractive range from which participants could choose. To facilitate students' understanding, myopia was assessed with the following question for each student: “Are you myopic?” The response categories were “yes” and “no”. If the student chose “yes,” he or she was asked to indicate the refractive range: low myopia (≤−0.5 to ≥ −3.0 D), moderate myopia (<−3.0 to>−6.0 D), and high myopia (≤−6.0 D). Every participant indicated whether he or she had uncorrected visual acuity or glasses-corrected visual acuity, with the response categories of “<1.0 (5.0)” and “≥1.0 (5.0)” (S1).

### 2.4. Statistical Analysis

All statistical analyses were performed using SPSS version 17.0 for Windows software (SPSS, Chicago, IL, USA). The data are presented as the number (%) or mean ± SD as appropriate. Analysis of variance or the chi-square test was used to compare the risk factors for myopia, including demographic (nonmodifiable) and behavioural (modifiable) characteristics. If the number of responses for some options was too small, multiple options were combined for the statistical analysis. The Pearson chi-square crosstab test was used for the single choice questions, and the Pearson chi-square for multiple responses was used for multiple choice questions. According to the chi-square test, the variables with statistical significance were selected for the logistic regression analysis. Adjusted odds ratios (ORs) and 95% confidence intervals (CIs) were determined. *p* values were 2-tailed, and <0.05 was considered statistically significant.

## 3. Results

### 3.1. Characteristics of the Study Population

Among the 4072 participants who returned questionnaires, 1831 (44.97%) participants did not meet the exclusion criteria, provided informed consent forms signed by their parents, and completed the questionnaires without obvious logical errors. These participants were included in the analysis for this study. In addition to 905 (22.22%) participants who were excluded based on the exclusion criteria, 1336 (32.81%) participants were excluded from the study for the following reasons: 915 (22.47%) participants had some missing information on the questionnaire (such as personal information or some response options), 232 (5.70%) participants did not return the informed consent form, and 189 (4.64%) participants were not granted permission to participate from their parents.

According to the questionnaire, there were 585 nonmyopic participants and 1246 myopic participants.  Table 1 shows the demographic characteristics of the study participants.

### 3.2. Factors with Significant Differences between the Myopia Group and the Nonmyopia Group

According to the Pearson chi-square crosstab test, there were significant differences in demographic characteristics between the nonmyopia group and the myopia group, including in age (*χ*2 = 53.030, *p* < 0.001), gender (*χ*2 = 21.956, *p* < 0.001), and parental myopia (myopic father: *χ*2 = 82.682, *p* < 0.001; myopic mother: *χ*2 = 112.49, *p* < 0.001) (Table 1). Regarding behavioural characteristics, there were significant differences between the two groups in some aspects of physical exercise and sleep. During the semester, average number of days per week participating in outdoor activities (*χ*2 = 6.864, *p*=0.009), average number of hours spent on each outdoor activity session (*χ*2 = 16.150, *p* < 0.001), average number of hours of sleep per night (*χ*2 = 22.093, *p* < 0.001), average night bedtime (*χ*2 = 20.636, *p* < 0.001), and average daily wake time (*χ*2 = 13.738, *p* < 0.001) were significantly different between the two groups. During holidays, significant differences were found between the two groups in the average number of hours spent on each outdoor activity session (*χ*2 = 24.048, *p* < 0.001), average night bedtime (*χ*2 = 42.891, *p* < 0.001), and average daily wake time (*χ*2 = 11.160, *p*=0.025) (Table 1).

**Figure 1:**
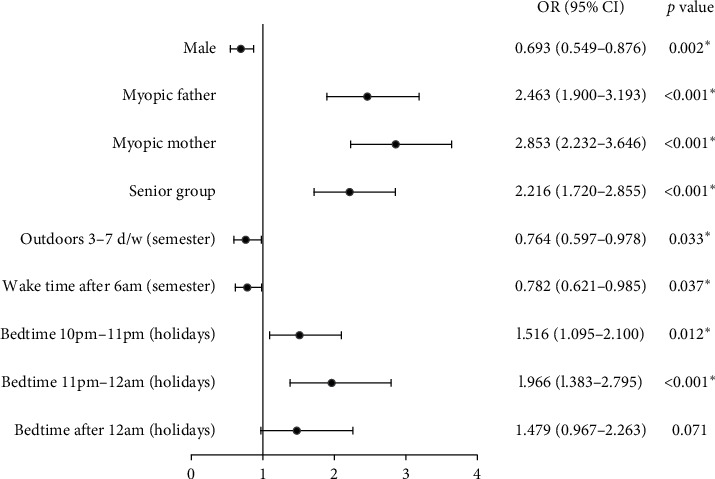
Associations between the myopia group and related factors: male gender, parental myopia, senior group, 3–7 days per week participating in outdoor activities during the semester (outdoors 3–7 d/w), a late daily wake time during the semester (wake time after 6 am), and a late bedtime during holidays. OR: odds ratio; CI: confidence interval. ^*∗*^Statistical significance (*p* < 0.05).

Compared with the nonmyopia group, the myopia group participants were more likely to be older (OR = 2.216, 95% CI: 1.720–2.855, *p* < 0.001), to have parents with myopia (myopic father: OR = 2.463, 95% CI: 1.900–3.193, *p* < 0.001; myopic mother: OR = 2.853 95% CI: 2.232–3.646, *p* < 0.001), and to have a late bedtime during holidays (before 10 pm: *p*=0.002; 10 pm-11 pm: OR = 1.516, 95% CI: 1.095–2.100, *p*=0.012; 11 pm-12 am: OR = 1.966, 95% CI: 1.383–2.795, *p* < 0.001; after 12 am: OR = 1.479, 95% CI: 0.967–2.263, *p*=0.017) but were less likely to be male (OR = 0.693, 95% CI: 0.549–0.876, *p*=0.002), to have a late daily wake time (OR = 0.782, 95% CI: 0.621–0.985, *p*=0.037), and to have more days per week participating in outdoor activities during the semester (OR = 0.764, 95% CI: 0.597–0.978, *p*=0.033) according to the logistic regression analysis (Table 1, Figure 1).

We inferred that more days per week participating in outdoor activities and a late daily wake time during the semester were inversely related to myopia, while a late bedtime during holidays was positively associated with myopia. Regarding demographic characteristics in our study, the proportion of nonmyopic adolescents was higher among males, while the proportion of myopic adolescents was higher among students with myopic parents and those aged 15–18 years.

### 3.3. Differences between the Adjusted Myopia Group and the Control Group

To eliminate the influence of gender, age, and parental myopia, we adjusted the number of cases in the myopia and nonmyopia groups for further research. Each group had the same number of cases with the same demographic characteristics. For example, if there were 20 senior high boys whose parents did not have myopia in the myopia group but 30 such boys in the nonmyopia group, 20 nonmyopic participants from the nonmyopia group with same characteristics were randomly selected as the control group and vice versa. For selection of the participants, a computer-generated list of random numbers was used. Thus, there were 521 nonmyopic participants in the control group and 521 myopic participants in the adjusted myopia group.

**Figure 2:**
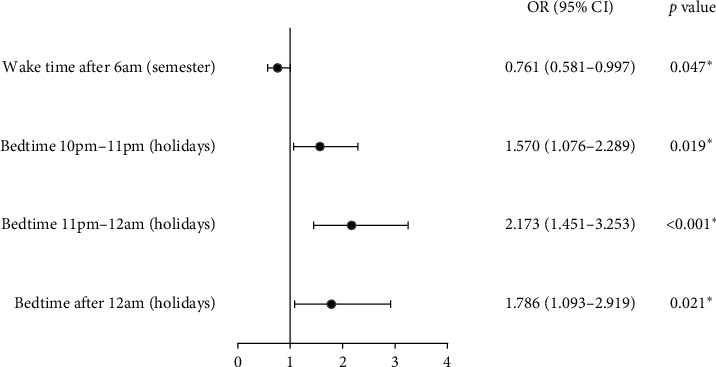
Associations between the adjusted myopia group and related factors: daily wake time after 6 am during the semester and a late bedtime during holidays. OR: odds ratio; CI: confidence interval. ^*∗*^Statistical significance (*p* < 0.05).

According to the Pearson chi-square crosstab test, there were significant differences between the two groups in average daily wake time (*χ*2 = 4.789, *p*=0.029) during the semester, the average number of hours spent on each outdoor activity session (*χ*2 = 3.175, *p*=0.004), and average night bedtime (*χ*2 = 14.506, *p*=0.002) during holidays (Table 2). Compared to the control group, the adjusted myopia group was more likely to have a late bedtime during holidays (before 10 pm: *p*=0.003; 10 pm-11 pm: OR = 1.570, 95% CI: 1.076–2.289, *p*=0.019; 11 pm-12 am: OR = 2.173, 95% CI: 1.451–3.253, *p* < 0.001; after 12 am: OR = 1.786, 95% CI: 1.093–2.919, *p*=0.021), but less likely to have a late daily wake time during the semester (OR = 0.761, 95% CI: 0.581–0.997, *p*=0.047) according to the logistic regression analysis (Table 2, Figure 2).

We inferred that a late daily wake time during the semester was inversely related to myopia, while a late bedtime during holidays was positively associated with myopia.

### 3.4. Differences in Sports Type between the Myopia Group and Nonmyopia Group

In multiple choice questions, we listed different kinds of sports (Table 3). Although the first three types of exercise were the same for the two groups, the order was different. Running was the first choice for the two groups, both for the semester and holidays. Basketball was the second choice in the nonmyopia group for both the semester and holidays, while it was the third choice in the myopia group. For both the semester and holidays, the percentages of students who practiced football, basketball, rope skipping, push-ups, and other options in the nonmyopia group were higher than those in the myopia group, but the percentages of students who engaged in table tennis, running, cycling, dance, and martial arts in the myopia group were higher than those in the nonmyopia group. According to the Pearson chi-square test for multiple responses, significant differences in sports type were identified between the two groups during the semester (*χ*2 = 65.619, *p* < 0.001) and holidays (*χ*2 = 27.525, *p*=0.016). However, there was no significant difference between the adjusted myopia group and the control group in sports type during the semester (*χ*2 = 21.254, *p*=0.095) or holidays (*χ*2 = 20.128, *p*=0.126).

## 4. Discussion

Our purpose in this study was to explore the correlation of myopia with physical exercise and sleep habits among adolescents. We analysed and compared adolescents' physical activities and sleep habits to evaluate the possible risk or protective factors that affected myopia among rural adolescents. We aimed to provide some feasible methods for intervening in adolescent myopia in the future.

In our study, myopia was found to be related to gender, age, and parental myopia among adolescents. Thirty-eight percent of the nonmyopia group was female, and 50% of the myopia group was female. The proportion of 15- to 18-year-old in the myopia group (77.53%) was higher than that in the nonmyopia group (61.20%). The proportion of students with myopic fathers in the myopia group was 39.09%, and the proportion with myopic mothers was 46.39%, which were higher than the percentages in the nonmyopia group (Table 1).According to a 10-year population-based survey in Beijing, girls were more likely than boys to have myopia, especially moderate myopia [14]. The North India Myopia Study (NIM Study) showed that the prevalence of myopia was also higher among girls and older (>11 years) children [15]. In addition, a study in Qingdao indicated that being older and having two myopic parents were risk factors for myopia [16]. Similarly, female gender, higher grade, and parental myopia were associated with an increased risk of myopia in children [17, 18]. In addition, other studies indicated that parental myopia was one of the risk factors for adolescents' myopia [19–22]. Regarding the demographic characteristics of our sample, in the myopia group, older age and parental myopia were risk factors for myopia, while male gender was a protective factor against myopia. These factors may be related to genetic and behavioural characteristics.

Previous studies on the behavioural characteristics of juvenile myopia have mainly focused on time spent in outdoor activities, distance and time of near work, sleep, and other possible factors such as light exposure time and electronic product usage. The result that increased outdoor exercise time was a protective factor of myopia is consistent with many studies [10, 21, 23–25]. Having at least 60 minutes of daily exercise [23] or one additional hour of outdoor time per week [26] could reduce the odds of a child having myopia. In addition, children who spent time outdoors at mid-day for 31–60 minutes or more than 60 minutes had better uncorrected visual acuity than students who spent less time outdoors [27]. However, other studies found that outdoor activities had less of a protective effect on myopia [6, 28]. A weak protective effect of the outdoor activity on myopia in Chinese rural children was observed in the Handan offspring myopia study [6]. The Anyang prospective cohort study revealed that time spent outdoors was not significantly associated with changes in refractive error or axial length among Chinese children aged 10–15 years [28]. In terms of behavioural characteristics, our results were slightly different from those of previous studies. Both before and after adjustment for demographic characteristics, there was a significant difference in the average time spent on each outdoor activity session during holidays between the two groups. However, in contrast to an outdoor activity duration of less than half an hour, various outdoor activity times longer than a half hour had no significant association with myopia. In addition, according to the logistic regression analysis, myopia was significantly correlated with the number of days participating in outdoor activities per week during the semester, but not significantly correlated with average time spent on each exercise session. There were significant differences between the two groups in sports type during the semester and holidays. However, after the demographic data adjustment, there was no significant correlation between the adjusted myopia group and the control group in the number of days per week participating in outdoor activities or sports type. Consistent with previous studies in China's suburbs, time and days participating in exercise may have little effect on juvenile myopia in Chinese suburban adolescents. For nonmyopic and low to moderate myopic Chinese suburban adolescents with corrected visual acuity of 1.0, our research suggested that the effect of physical exercise time and type on myopia was not obvious, which may be related to the participants in the myopia group not having high myopia.

Moreover, we also studied the association between sleep habits and myopia in adolescents. A study of Korean adolescents provided population-based epidemiologic evidence of an inverse relationship between sleep duration and myopia, while high myopia was not associated with sleep duration [11]. However, sleep quality in Japanese children was significantly correlated with myopic error, especially in the high myopia group. High myopic error was significantly correlated with poor Pittsburgh Sleep Quality Index (PSQI) scores, short sleep duration, and late bedtime [12]. In contrast, the association between sleep duration and myopia (with a range of –8.25 to + 0.38 D) was not significant for total (night + mid-day) sleep, but odds of myopia increase were associated with more night-time sleep among Chinese children in Guangzhou [13]. Our study demonstrated that, according to the logistic regression analysis, myopia was not statistically associated with sleep duration during the semester and holidays. This may be related to our myopic participants not having high myopia and not having a corrected visual acuity less than 1.0. However, differences in other sleep habits were found between the myopia group and the nonmyopia group in our study. Both before and after the data adjustment, myopia was positively associated with late bedtime during holidays but negatively correlated with daily wake time after 6 am during the semester. We inferred that a daily wake time after 6 am during the semester and a late bedtime during holidays may have greater effects on low to moderate myopia than sleep duration among suburban adolescents. Changing children's habits for wake time during the semester and bedtime during holidays may be two feasible follow-up methods for future myopia intervention in suburban adolescents.

The present study had several limitations. First, despite the diversity of schools, the data were based on findings from some schools of one suburban district (Qingpu, Shanghai); hence, the results may not be universally representative. Second, 1831 valid data points in this study were selected from 4072 questionnaires, accounting for approximately 44.97% of the total number of data points. In addition to data excluded due to participants meeting the exclusion criteria and parents disapproving of their children's participation in the study, some questionnaire data with missing options or logical errors were excluded as well. The excluded questionnaire data might have resulted in the exclusion of some students who did not know their refractive range because they were informed of their abnormal vision for the first time at the most recent student medical examination or some students whose parents neglected to take them for an optometry examination and follow-up. In addition, because the student medical examination did not include cycloplegic refraction, students chose the diopter of their own glasses. We note that this research concentrated only on the participants' refractive range, not the precise myopic diopter, and low and moderate myopia with corrected visual acuity of 1.0 or more, excluding corrected visual acuity of less than 1.0. Moreover, the use of subjective questionnaire data may have caused slight bias. Additionally, the exercise and sleep time of the students indicated might have been their routine time, rather than the average time, but even the routine time would have reflected students' lifestyles.

## 5. Conclusion

Despite these limitations, our paper is the first to examine the association of wake time and bedtime, both of which appear to be changeable habits among adolescents, with myopia. Additionally, the association of days and time participating in outdoor exercise with low to moderate myopia among Chinese suburban adolescents does not seem to be as great as that of sleep habits. Having children wake up after 6 am during the semester and go to bed before 10 pm during holidays may be two feasible ways to intervene in juvenile myopia in the future. Future research could seek to provide consistent evidence of a potential association between myopia and sleep habits.

## Figures and Tables

**Table 1:** Demographic characteristics of the study population and the factors of the myopia and nonmyopia groups during the semester and holidays.

	Nonmyopia	Myopia	*χ* ^2^	*p* (chi-square test)	*p* (logistic regression analysis)	OR	95% CI	
	(*N* = 585)	(*N* = 1246)					Lower	Upper
Gender, male, *N* (%)	361 (61.71)	623 (50.00)	21.956	**<0.001** ^*∗*^	**0.002** ^*∗*^	**0.693**	0.549	0.876
Parental myopia								
Father, *N* (%)	104 (17.78)	487 (39.09)	82.682	**<0.001** ^*∗*^	**<0.001** ^*∗*^	**2.463**	1.900	3.193
Mother, *N* (%)	118 (20.17)	578 (46.39)	112.490	**<0.001** ^*∗*^	**<0.001** ^*∗*^	**2.853**	2.232	3.646
Age (mean ± SD, years)	14.53 ± 1.66	15.10 ± 1.21	53.030	**<0.001** ^*∗*^				
Junior group (11–14 years), *N* (%)	227 (38.80)	280 (22.47)						
Senior group (15–18 years), *N* (%)	358 (61.20)	966 (77.53)			**<0.001** ^*∗*^	**2.216**	1.720	2.855
During the semester								
Average number of days per week participating in outdoor activities, *N* (%)			6.864	**0.009** ^*∗*^				
≤2 days	271 (46.32)	659 (52.89)						
3–7 days	314 (53.68)	587 (47.11)			**0.033** ^*∗*^	**0.764**	0.597	0.978
Average number of hours spent on each outdoor activity session, *N* (%)			16.150	**<0.001** ^*∗*^				
<Half an hour	149 (25.47)	328 (26.32)			0.139			
≥Half an hour and < one hour	253 (43.25)	634 (50.88)			0.198	1.212	0.904	1.624
≥One hour	183 (31.28)	284 (22.80)			0.753	0.946	0.671	1.334
Average number of hours per week of outdoor activities, *N* (%)			0.016	0.900				
<3 hours	315 (53.85)	667 (53.53)						
≥3 hours	270 (46.15)	579 (46.47)			0.046^*∗*^	1.320	1.006	1.732
Average number of hours per week of indoor activities, *N* (%)			1.468	0.226				
<3 hours	464 (79.32)	1018 (81.70)						
≥3 hours	121 (20.68)	228 (18.30)			0.925	0.985	0.713	1.359
Mid-day nap, *N* (%)			2.440	0.118				
Yes	142 (24.27)	262 (21.03)			0.068	0.781	0.598	1.019
No	443 (75.73)	984 (78.97)						
Average number of hours per night of sleep, *N* (%)			22.093	**<0.001** ^*∗*^				
<6 hours	98 (16.75)	218 (17.50)			0.260			
≥6 hours and <8 hours	371 (63.42)	883 (70.87)			0.188	1.249	0.897	1.739
≥8 hours	116 (19.83)	145 (11.63)			0.879	1.035	0.666	1.608
Average night bedtime, *N* (%)			20.636	**<0.001** ^*∗*^				
Before 10 pm (excluding 10 pm)	220 (37.61)	338 (27.13)			0.775			
10 pm-11 pm (excluding 11 pm)	244 (41.71)	607 (48.71)			0.996	1.001	0.756	1.326
After 11 pm (including 11 pm)	121 (20.68)	301 (24.16)			0.554	1.118	0.773	1.617
Average daily wake time, *N* (%)			13.738	**<0.001** ^*∗*^				
Before 6 am (excluding 6 am)	229 (39.15)	603 (48.39)						
After 6 am (including 6 am)	356 (60.85)	643 (51.61)			**0.037** ^*∗*^	**0.782**	0.621	0.985
During holidays								
Average number of days per week participating in outdoor activities, *N* (%)			0.007	0.933				
≤2 days	381 (65.13)	809 (64.93)						
3–7 days	204 (34.87)	437 (35.07)			0.136	1.214	0.941	1.566
Average number of hours spent on each outdoor activity session, *N* (%)			24.048	**<0.001** ^*∗*^				
<Half an hour	128 (21.88)	277 (22.23)			0.035^*∗*^			
≥Half an hour and < one hour	144 (24.62)	373 (29.94)			0.742	1.056	0.762	1.464
≥One hour and <2 hours	144 (24.62)	359 (28.81)			0.507	1.129	0.789	1.615
≥2 hours	169 (28.88)	237 (19.02)			0.096	0.719	0.487	1.061
Average number of hours per week of outdoor activities, *N* (%)			0.760	0.383				
<3 hours	268 (45.81)	598 (47.99)						
≥3 hours	317 (54.19)	648 (52.01)			0.521	0.909	0.680	1.216
Average number of hours per week of indoor activities, *N* (%)			0.255	0.614				
<3 hours	414 (70.77)	896 (71.91)						
≥3 hours	171 (29.23)	350 (28.09)			0.469	1.114	0.832	1.492
Mid-day nap, *N* (%)			1.938	0.164				
Yes	168 (28.72)	398 (31.94)			0.063	1.259	0.987	1.605
No	417 (71.28)	848 (68.06)						
Average number of hours per night of sleep, *N* (%)			4.579	0.205				
<6 hours	46 (7.86)	83 (6.66)			0.892			
≥6 hours and <8 hours	169 (28.90)	415 (33.31)			0.873	1.039	0.650	1.661
≥8 hours and <10 hours	281 (48.03)	584 (46.87)			0.740	1.081	0.682	1.715
≥10 hours	89 (15.21)	164 (13.16)			0.503	1.198	0.705	2.035
Average night bedtime, *N* (%)			42.891	**<0.001** ^*∗*^				
Before 10 pm (excluding 10 pm)	155 (26.50)	188 (15.09)			**0.002** ^*∗*^			
10 pm-11 pm (excluding 11 pm)	172 (29.40)	422 (33.87)			**0.012** ^*∗*^	**1.516**	1.095	2.100
11 pm-12 am (excluding 12 am)	150 (25.64)	438 (35.15)			**<0.001** ^*∗*^	**1.966**	1.383	2.795
After 12 am (including 12 am)	108 (18.46)	198 (15.89)			0.071	1.479	0.967	2.263
Average daily wake time, *N* (%)			11.160	**0.025** ^*∗*^				
Before 7 am (excluding 7 am)	100 (17.09)	168 (13.49)			0.165			
7 am-8 am (excluding 8 am)	121 (20.68)	316 (25.36)			0.059	1.423	0.986	2.053
8 am-9 am (excluding 9 am)	142 (24.27)	320 (25.68)			0.555	1.120	0.770	1.629
9 am-10 am (excluding 10 am)	107 (18.30)	244 (19.58)			0.225	1.284	0.857	1.925
After 10 am (including 10 am)	115 (19.66)	198 (15.89)			0.819	0.950	0.615	1.469

^*∗*^Statistical significance (*p* < 0.05).

**Table 2:** Demographic characteristics of the study population and the factors of adjusted myopia and control groups during the semester and holidays after adjustment.

	Control	Adjusted myopia	*χ* ^2^	*p* (chi-square test)	*p* (logistic regression analysis)	OR	95% CI	
	(*N* = 521)	(*N* = 521)					Lower	Upper
Gender, male, *N* (%)	317 (60.84)	317 (60.84)	0.000	1.000	0.506	1.098	0.834	1.444
Parental myopia								
Father, *N* (%)	104 (19.96)	104 (19.96)	0.000	1.000	0.920	1.017	0.737	1.402
Mother, *N* (%)	118 (22.65)	118 (22.65)	0.000	1.000	0.686	0.939	0.692	1.274
Age (mean ± SD years)	14.67 ± 1.66	14.84 ± 1.35	0.000	1.000				
Junior group (11–14 years), *N* (%)	163 (31.29)	163 (31.29)						
Senior group (15–18 years), *N* (%)	358 (68.71)	358 (68.71)			0.621	0.927	0.688	1.251
During the semester								
Average number of days per week participating in outdoor activities, *N* (%)			3.230	0.072				
≤2 days	241 (46.26)	270(51.82)						
3–7 days	280 (53.74)	251 (48.18)			0.017	0.698	0.519	0.939
Average number of hours spent on each outdoor activity session, *N* (%)			5.507	0.064				
<Half an hour	140 (26.87)	128 (24.57)			0.220			
≥Half an hour and < one hour	217 (43.25)	254 (48.75)			0.224	1.239	0.877	1.751
≥One hour	164 (31.48)	139 (26.68)			0.892	0.972	0.650	1.455
Average number of hours per week of outdoor activities, *N* (%)			0.467	0.494				
<3 hours	285 (54.70)	274 (52.59)						
≥3 hours	236 (45.30)	247 (47.41)			0.073	1.338	0.973	1.841
Average number of hours per week of indoor activities, *N* (%)			0.601	0.438				
<3 hours	412 (79.08)	422 (81.00)						
≥3 hours	109 (20.92)	99 (19.00)			0.530	0.887	0.611	1.289
Mid-day nap, *N* (%)			0.628	0.428				
Yes	133 (25.53)	122 (23.42)			0.457	0.890	0.654	1.210
No	388 (74.47)	399 (76.58)						
Average number of hours per night of sleep, *N* (%)			5.312	0.070				
<6 hours	95 (18.24)	78 (14.97)			0.242			
≥6 hours and <8 hours	331 (63.53)	366 (70.25)			0.114	1.370	0.927	2.026
≥8 hours	95 (18.23)	77 (14.78)			0.524	1.183	0.705	1.984
Average night bedtime, *N* (%)			1.587	0.452				
Before 10 pm (excluding 10 pm)	191 (36.66)	177 (33.97)			0.799			
10 pm-11 pm (excluding 11 pm)	213 (40.88)	233 (44.72)			0.532	0.901	0.650	1.249
After 11 pm (including 11 pm)	117 (22.46)	111 (21.31)			0.896	0.972	0.634	1.490
Average daily wake time, *N* (%)			4.789	**0.029** ^*∗*^				
Before 6 am (excluding 6 am)	208 (39.92)	243 (46.64)						
After 6 am (including 6 am)	313 (60.08)	278 (53.36)			**0.047** ^*∗*^	**0.761**	0.581	0.997
During holidays								
Average number of days per week participating in outdoor activities, *N* (%)			0.346	0.556				
≤2 days	348 (66.79)	339 (65.07)						
3–7 days	173 (33.21)	182 (34.93)			0.254	1.189	0.883	1.600
Average number of hours spent on each outdoor activity session, *N* (%)			13.175	**0.004** ^*∗*^				
<Half an hour	119 (22.84)	104 (19.96)			**0.015** ^*∗*^			
≥Half an hour and < one hour	130 (24.95)	138 (26.49)			0.490	1.146	0.778	1.690
≥One hour and <2 hours	122 (23.42)	166 (31.86)			0.054	1.516	0.993	2.317
≥2 hours	150 (28.79)	113 (21.69)			0.522	0.861	0.543	1.364
Average number of hours per week of outdoor activities, *N* (%)			0.035	0.852				
<3 hours	241 (46.26)	244 (46.83)						
≥3 hours	280 (53.74)	277 (53.17)			0.338	0.845	0.599	1.192
Average number of hours per week of indoor activities, *N* (%)			0.115	0.734				
<3 hours	370 (71.02)	365 (70.06)						
≥3 hours	151 (28.98)	156 (29.94)			0.287	1.199	0.858	1.676
Mid-day nap, *N* (%)			0.042	0.837				
Yes	148 (28.41)	151 (28.98)			0.716	1.055	0.791	1.406
No	373 (71.59)	370 (71.02)						
Average number of hours per night of sleep, *N* (%)			1.496	0.683				
<6 hours	43 (8.25)	41 (7.87)			0.947			
≥6 hours and <8 hours	157 (30.13)	165 (31.67)			0.686	0.896	0.525	1.528
≥8 hours and <10 hours	241 (46.26)	248 (47.60)			0.934	0.978	0.579	1.653
≥10 hours	80 (15.36)	67 (12.86)			0.932	0.974	0.534	1.777
Average night bedtime, *N* (%)			14.506	**0.002** ^*∗*^				
Before 10 pm (excluding 10 pm)	138 (26.49)	97 (18.62)			**0.003** ^*∗*^			
10 pm-11 pm (excluding 11 pm)	150 (28.79)	155 (29.75)			**0.019** ^*∗*^	**1.570**	1.076	2.289
11 pm-12 am (excluding 12 am)	134 (25.72)	180 (34.55)			**<0.001** ^*∗*^	**2.173**	1.451	3.253
After 12 am (including 12 am)	99 (19.00)	89 (17.08)			**0.021** ^*∗*^	**1.786**	1.093	2.919
Average daily wake time, *N* (%)			7.736	0.102				
Before 7 am (excluding 7 am)	90 (17.28)	76 (14.59)			0.154			
7 am-8 am (excluding 8 am)	108 (20.73)	133 (25.53)			0.105	1.417	0.929	2.161
8 am-9 am (excluding 9 am)	126 (24.18)	131 (25.14)			0.898	1.029	0.665	1.592
9 am-10 am (excluding 10 am)	94 (18.04)	103 (19.77)			0.604	1.132	0.709	1.808
After 10 am (including 10 am)	103 (19.77)	78 (14.97)			0.410	0.805	0.481	1.348

^*∗*^Statistical significance (*p* < 0.05).

**Table 3:** Differences in sports type between the nonmyopia group and the myopia group.

Kind of sports	Nonmyopia (*N* = 585) (%)		Myopia (*N* = 1246) (%)	
	Semester	Holidays	Semester	Holidays
A. Football	13.4^*∗*^	8.4^*∗*^	10.8	5.7
B. Basketball	33.4^*∗*^	26.0^*∗*^	26.3	22.3
C. Badminton	28.3	18.5^*∗*^	29.4^*∗*^	18.3
D. Volleyball	0.9	1.0^*∗*^	1.6^*∗*^	0.4
E. Table tennis	11.0	3.8	13.4^*∗*^	4.4^*∗*^
F. Running	39.2	32.1	46.9^*∗*^	39.5^*∗*^
G. Swimming	0.7^*∗*^	8.0	0.5	9.5^*∗*^
H. Rope skipping	4.6^*∗*^	6.8^*∗*^	2.2	5.6
I. Cycling	5.3	23.2	6.0^*∗*^	24.2^*∗*^
J. Dance	1.7	4.6	4.8^*∗*^	5.5^*∗*^
K. Martial arts	2.1	0.3	7.0^*∗*^	1.1^*∗*^
L. Sit ups	11.1^*∗*^	14.0	10.5	16.4^*∗*^
M. Push-ups	8.9^*∗*^	10.1^*∗*^	7.1	9.8
N. Others	6.2^*∗*^	9.1^*∗*^	6.1	8.4

^*∗*^The students who participated in the sport at a higher percentage between the two groups during the semester or holidays.

## Data Availability

The data used to support the findings of this study are included within the article.

## Supplementary Materials

Supplementary Materials S1: questionnaire on the correlation of myopia with physical exercise and sleep habits among adolescents. The questionnaire contains sociodemographic information and relevant behavioural factors of the study.
