# Supplementary material for: Correlation of Myopia with Physical Exercise and Sleep Habits among Suburban Adolescents
Source: J Ophthalmol. 2020 Jul 25;2020:2670153. doi: 10.1155/2020/2670153 (PMC7399760; doi:10.1155/2020/2670153)
Supplement: Supplementary Materials — S1: questionnaire on the correlation of myopia with physical exercise and sleep habits among adolescents. The questionnaire contains sociodemographic information and relevant behavioural factors of the study. [file 2670153.f1.pdf]

Questionnaire on the Correlation of Myopia with Physical Exercise and Sleep Habits among Adolescents

Full name： Gender：male/female Class： Student ID:

Please fill in the results in brackets.

1. Date of birth: （ ）

2. Is your father (or mother) myopic? A No B Yes

Father（ ） Mother（ ）

3. Are you myopic? A No B Yes（Choose your refractive range）

For example：A，B-C，B-D，B-E

C. ≤-0.5 to ≥-3.0 diopters D. <-3.0 to>-6.0 diopters E.≤-6.0 diopters

Right eye（ ） Left eye（ ）

4. What is your eyesight without glasses? Right eye（ ） Left eye（ ）

What is your eyesight when you wear glasses? (Do not fill in if there is no myopia.) Right eye（ ） Left eye（ ）

A. ≥1.0（5.0） B. < 1.0（5.0）

（ ） 5. Do you have any of the following eye diseases?

A.None B. Hyperopia C. Amblyopia D. Astigmatism E. Others (please indicate)( )

6. How many days do you participate in outdoor activities every week on average?

（ ） 1）During the semester

（ ） 2）During holidays

A.0 day B.1-2 days C.3-4 days D.5-6 days E.7 days

7. How long do you spend on outdoor activities during every activity session on average?

（ ） 1）During the semester

（ ） 2）During holidays

A. < half an hour B. ≥ half an hour and < one hour

C. ≥ one hour and < two hours D. ≥ two hours

8. What kinds of sports do you usually do?（Select 3 items at most）

（ ） 1）During the semester

（ ） 2）During holidays

A. Football	D. Volleyball	G. Swimming	J. Dance	M. Push ups
B. Basketball	E. Table tennis	H. Rope skipping	K. Martial arts	N. Others(please indicate)
C. Badminton	F. Running	I. Cycling	L. Sit ups	

9. How much time do you spend on outdoor activities every week on average?

（ ） 1）During the semester

(　　) 2）During holidays

A. < 3 hours　　B. ≥ 3 hours and < 6 hours

C. ≥ 6 hours and < 12 hours　　D. ≥ 12 hours

10. How much time do you spend on indoor activities on average every week?

(　　) 1）During the semester

(　　) 2）During holidays

A. < 3 hours　　B. ≥ 3 hours and < 6 hours

C. ≥ 6 hours and < 12 hours　　D. ≥ 12 hours

11. Do you have a habit of taking midday naps?　A.Yes　B.No

(　　) 1）During the semester

(　　) 2）During holidays

12. How long do you sleep every night on average?

(　　) 1）During the semester

(　　) 2）During holidays

A. < 6 hours　　B. ≥ 6 hours and < 8 hours

C. ≥ 8 hours and < 10 hours　　D. ≥ 10 hours

13. When do you go to bed every night on average?

(　　) 1）During the semester

(　　) 2）During holidays

A. Before 8pm(excluding 8pm)　B. 8pm-9pm (excluding 9pm)　C.9pm-10pm(excluding 10pm)

D. 10pm-11pm(excluding 11pm)　E. 11pm-12am (excluding 12am)　F. After 12am(including 12am)

14. What time do you get up every day on average?

(　　) 1）During the semester

(　　) 2）During holidays

A. Before 6am(excluding 6am)　B.6am-7am(excluding 7am)　C.7am-8am(excluding 8am)　D. 8am-9am(excluding 9am)

E. 9am-10am(excluding 10am)　F.10am-11am(excluding 11am)　G.11am-12pm(excluding 12pm)　H. After 12pm(including 12pm)
